# Meta-analysis Driven Strain Design for Mitigating Oxidative Stresses Important in Biomanufacturing

**DOI:** 10.1021/acssynbio.3c00572

**Published:** 2024-06-27

**Authors:** PV Phaneuf, SH Kim, K Rychel, C Rode, F Beulig, BO Palsson, L Yang

**Affiliations:** † Novo Nordisk Foundation Center for Biosustainability, Technical University of Denmark, Kemitorvet, Building 220. Kongens Lyngby 2800, Denmark; ‡ Department of Bioengineering, University of California, San Diego, La Jolla ,California92093-0412 ,United States; § Bioinformatics and Systems Biology Program, University of California, San Diego, La Jolla ,California92093-0021, United States; ∥ Department of Pediatrics, University of California, San Diego, La Jolla ,California 92093-0412, United States

**Keywords:** ALE mutations, iModulons, meta-analysis driven strain design, reactive oxygen species, acid stress, SOS response

## Abstract

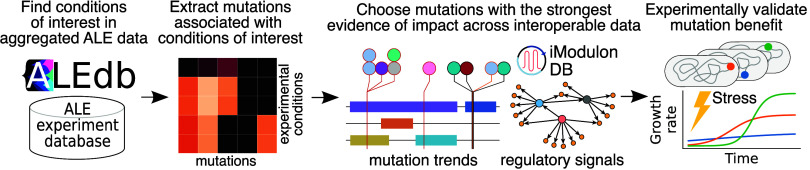

As the availability of data sets increases, meta-analysis leveraging aggregated and interoperable data types is proving valuable. This study leveraged a meta-analysis workflow to identify mutations that could improve robustness to reactive oxygen species (ROS) stresses using an industrially important melatonin production strain as an example. ROS stresses often occur during cultivation and negatively affect strain performance. Cellular response to ROS is also linked to the SOS response and resistance to pH fluctuations, which is important to strain robustness in large-scale biomanufacturing. This work integrated more than 7000 *E. coli* adaptive laboratory evolution (ALE) mutations across 59 experiments to statistically associate mutated genes to 2 ROS tolerance ALE conditions from 72 unique conditions. Mutant *oxyR*, *fur*, *iscR*, and *ygfZ* were significantly associated and hypothesized to contribute fitness in ROS stress. Across these genes, 259 total mutations were inspected in conjunction with transcriptomics from 46 iModulon experiments. Ten mutations were chosen for reintroduction based on mutation clustering and coinciding transcriptional changes as evidence of fitness impact. Strains with mutations reintroduced into *oxyR*, *fur*, *iscR*, and *ygfZ* exhibited increased tolerance to H_2_O_2_ and acid stress and reduced SOS response, all of which are related to ROS. Additionally, new evidence was generated toward understanding the function of *ygfZ*, an uncharacterized gene. This meta-analysis approach utilized aggregated and interoperable multiomics data sets to identify mutations conferring industrially relevant phenotypes with the least drawbacks, describing an approach for data-driven strain engineering to optimize microbial cell factories.

## Introduction

Strain robustness is critical for microbial cell factory development and biobased manufacturing of chemicals or protein products. Many external and internal stresses may adversely affect the strain performance. One such stress is oxidative stress, which often negatively impacts strain robustness during fermentation, especially during large-scale cultivations. Reactive oxygen species (ROS), including H_2_O_2_, superoxide anion radicals, and hydroxyl radicals, are toxic byproducts naturally generated during aerobic growth. ROS stress often occurs when oxygen levels increase in reactors; when toxic substrates, intermediates, or products accumulate; or when NADPH depletes.^1−3^ When accumulated intracellularly, ROS can damage DNA, metalloproteins, and many other cellular processes.^3^

Microorganisms have developed various mechanisms to prevent ROS damage. One such mechanism is the ROS scavenging and DNA/protein damage repair system, regulated by the OxyR (oxidative stress regulator) transcription factor (TF).^4^ The iron uptake system can also be modified to prevent excess iron-related ROS production from Fenton reactions, regulated by the Fur (ferric uptake regulator) TF.^4,5^ Another is a system for the maintenance and assembly of iron–sulfur (Fe–S) clusters, which are essential for many cellular processes and are affected by ROS^6^ and regulated by the IscR (iron–sulfur cluster regulator) TF.

Severe ROS stress can lead to DNA damage through various mechanisms, including guanine oxidative lesions.^7^ ROS stress can trigger the SOS response, a set of cellular reactions to DNA damage. Strains that have elevated levels of SOS induction for an extended period may have decreased genetic stability and loss of the desired production in fermentation.^8^ SOS response levels can be an important indicator of strain robustness during fermentation scale-up.

Acid stress is another stress that might occur in fermentation. For example, in large-scale bioreactors, slow mixing of the base might lead to a pH fluctuation. Acetic acid and amino acid accumulation could create a transient local low pH environment unfavorable in bioprocesses due to its negative impact on growth and production. Several studies have demonstrated the link between acid and ROS stress, and engineered strains with reduced intracellular ROS can better survive at low pH.^9−12^ One of the reasons could be that Fe–S clusters are labile to acid stress.^13,14^

The rational design of strains for tolerance to adverse conditions is generally challenging due to the variety and complexity of systems involved, including multiple stress response pathways and cellular processes that collectively determine the organism’s resilience and adaptability.^15−18^ Adaptive laboratory evolution (ALE) is an experimental evolution method that has the potential to provide novel solutions to strain design in the form of mutations^19−22^ and has been previously used to find strain design solutions for growth rate optimization, enhanced stress tolerance, substrate utilization, increased product titer/yield, and general discovery.^19−25^ It has also been observed that ALE-generated mutations are not frequently observed in natural isolates,^26^ highlighting their distinctiveness. It is hypothesized that an individual ALE experiment selects mutations that are readily accessible and sufficiently effective, though not necessarily the most potent.^27^ Experimental evidence further emphasizes this potential by demonstrating that different substitutions to a single gene can induce a phenotype across a range of intensities.^28^ ALEdb (aledb.org), a publicly accessible database of aggregated ALE mutations,^29^ has the potential to provide data that could lead to a more comprehensive understanding of gain-of-function mutations and result in a more effective strain design solution.^30,31^

Additional resources that are interoperable with ALEdb data are available for describing the consequences of ALE mutations, ultimately providing insights into which ALE mutations may be the most effective for a desired phenotype. iModulons represent independently modulated gene sets computed from a large compendium of transcriptomic data, and their activities represent the abundance of coregulated gene products in specific conditions.^32^ iModulons have proven valuable in broadly elucidating microbial transcriptional regulator networks^33−42^ and describing systems-level changes in a strain according to experimental conditions.^4,5,43−46^ Comprehensive iModulon data are available through a publicly accessible database, iModulonDB (imodulondb.org).^33,47^ The combination of mutations and corresponding iModulon activity level changes in ALE strains has proven informative toward understanding systems-level changes brought about by ALE mutations.^4,43,44,46,48^ The combination of ALE mutations and iModulon activities could also focus mutation screening efforts on the subset of mutations with the most promise of rendering phenotypes of interest and a minimum of side effects. A recent study characterized mutations and iModulons from ALE strains that tolerated paraquat, a ROS-inducing agent, proposing some interesting hypothetical mechanisms that were not directly validated.^5^

An *E. coli* strain producing melatonin has been previously developed.^49^ This study demonstrated that this strain has elevated ROS stress compared to the nonengineered ancestor strain. Consequently, it also has a higher SOS response and a decreased tolerance to acid stress. To address this challenge, a meta-analysis workflow was employed utilizing aggregated ALE mutations from 59 different experiments and interoperable transcriptomic data from 46 iModulon experiments. This approach predicted a small set of mutations that mitigate oxidative stress, and their effects were experimentally validated.

## Results

### Parent Strain Has Elevated ROS Stress and SOS Response

**Figure 1:**
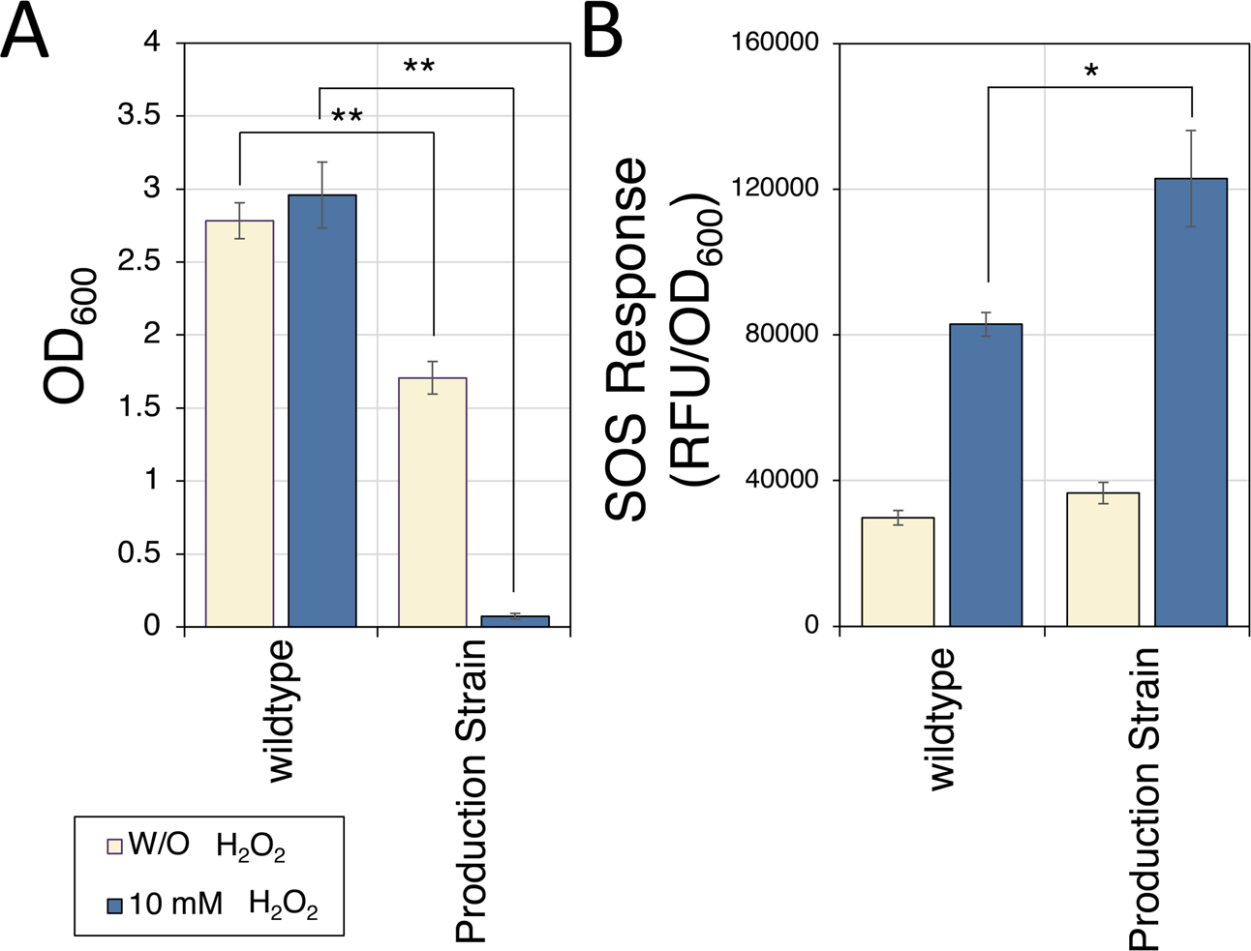
The parent strain has higher ROS stress sensitivity and SOS response compared to the wild type. (A) Growth of the wild-type strain and melatonin production strain (parent strain) with or without H_2_O_2_. The wild-type strain could tolerate 10 mM H_2_O_2_, but the parent strain cannot. (B) SOS response of the wild-type and parent strain measured by a GFP-based SOS sensor. The parent strain has a slightly higher SOS response when growing in the LB medium and about 50% higher SOS response level in the presence of H_2_O_2_ (*p < 0.05, **p < 0.001). See Materials and Methods for details.

We have previously engineered an *E. coli* strain producing melatonin by expressing heterologous enzymes required for melatonin synthesis from tryptophan,^49^ as well as genome engineering for improving tryptophan synthesis from glucose (Table S1^50^). Because factors like product toxicity, acid accumulation, and heterologous protein expression often lead to increased ROS stress,^10,51^ the ROS stress level was tested by growing the strain in H_2_O_2_. The melatonin production strain HMP3427 (parent strain) cannot grow in the presence of 10 mM H_2_O_2_ in minimal media after 72 h, but the wild-type strain grew to a similar OD with or without H_2_O_2_ (Figure 1A). This suggests that it is more sensitive to H_2_O_2_ than the wild type.

We further tested the SOS response level in the parent because ROS stress can lead to elevated DNA damage and SOS response. Using a previously reported SOS biosensor,^52^ the production (parent) strain demonstrated a slightly higher SOS response in normal growth conditions (Figure 1B). When treated with H_2_O_2_, the difference in SOS response levels between the parent strain and wild type increased dramatically. These results suggest that the production strain has a high level of ROS stress and related SOS response compared to the wild type. A data-driven approach was then applied to design mutations aimed at mitigating ROS stress and improving strain robustness.

### A Meta-analysis of ALE Experiment Mutations Revealed Mutant Genes for Potential ROS Tolerance

To identify mutations that could render ROS tolerance to a host, a meta-analysis was performed on ALE experiment mutations in ALEdb, their conditions, and their potential impact. First, ALE-mutated genetic features (genes or intergenic regions) were statistically associated with ALE conditions involving ROS stress to identify what mutated features could contribute to ROS tolerance (7670 public mutations across 72 unique conditions from 59 ALE experiments). ALE mutation data were exported from aledb.org and were described by their sequence changes and the conditions in which they were manifested.^29^ Second, to investigate the potential impact of individual mutations on a host’s phenotype, both mutation clustering on genetic features and changes in gene expression were examined. Individual mutations on genetic features of interest were investigated for their potential impact according to mutation clustering on the amino acid sequence and protein structure. Transcriptional changes were investigated through iModulon data and were acquired from a combination of data exported from iModulonDB’s already available *E. coli* data set^33,47^ and from new samples generated with this study for a total of 46 iModulon experiments. Finally, a small subset of mutations for each genetic feature of interest was chosen for reintroduction and tested for increased tolerance.

**Figure 2:**
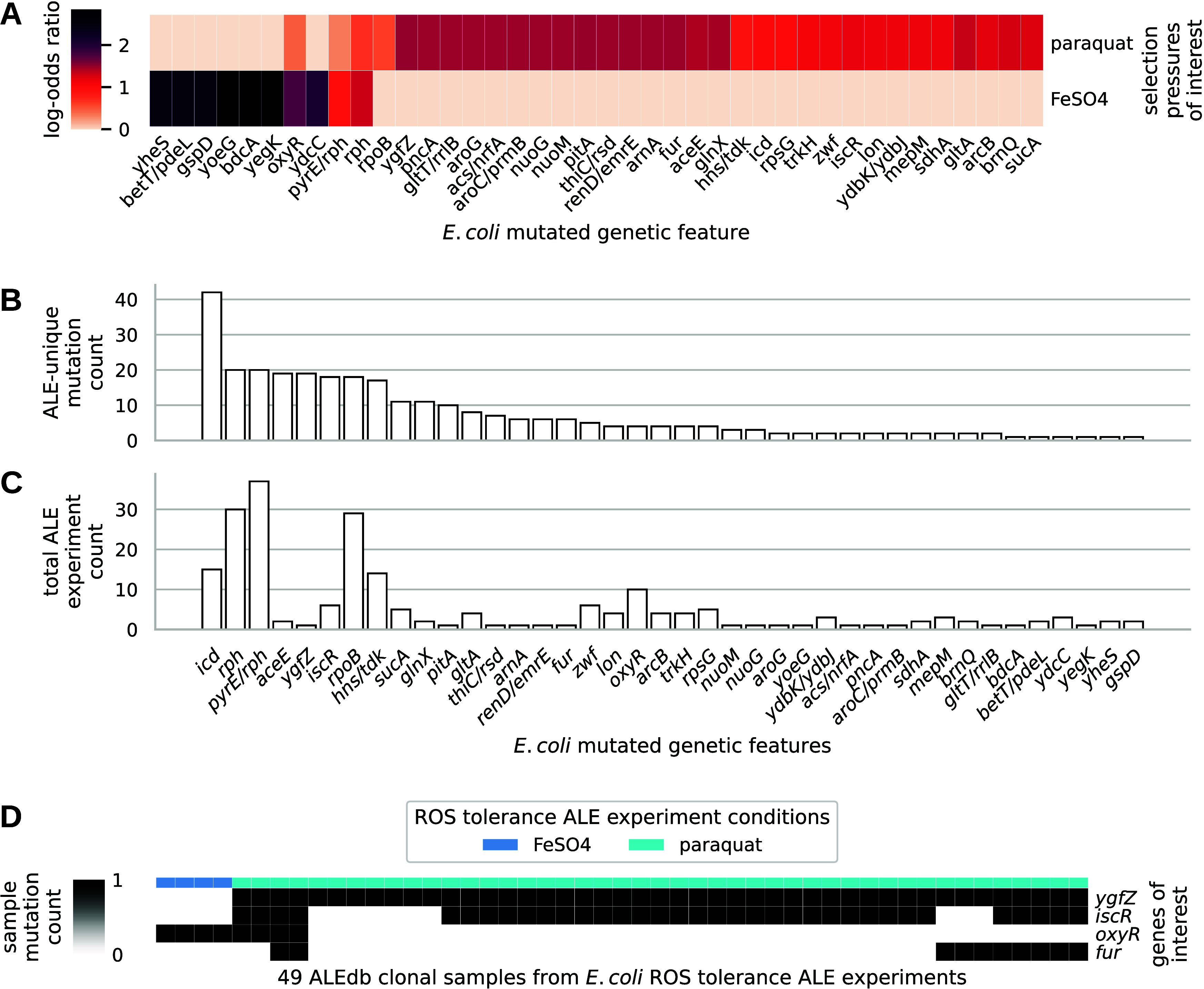
Meta-analysis of mutated genetic features and their experimental conditions in ALEdb samples. (A) Public ALEdb mutated genetic features statistically associated with sources of ROS stress in ALEdb (Fisher’s exact test, *p* value < 0.01, Bonferroni corrected). (B) The features sorted according to the sum of their unique mutations per independent ALE replicate across paraquat and FeSO4 ALE experiments. The total amount of ALE experiments per feature also displayed. (C) The mutation count of genes of interest in samples for ALE experiments explicitly involving ROS stresses. Slight differences in totals exist between B and C and Figures 3 through 6 due to different filtering methods applied (see the ALEdb Mutations section).

ALEdb’s conditions revealed 40 mutant genetic features significantly associated with ROS stress (Figure 2A). Of these, three were transcription factors—*oxyR*, *iscR*, and *fur*—linked to one or more iModulons known to participate in ROS stress^5^ and are likely to have a significant impact on the host’s phenotype as they regulate the expression of numerous other genes.^33^ Additionally, mutant *oxyR* may hold potential for general ROS tolerance according to its significant association with both ROS ALEdb conditions available. The amount of ALE-unique mutations to genetic features in ROS ALEdb experiments can be used as potential evidence of a mutant feature’s fitness benefit (Figure 2B). From this, many of the less frequently mutated features can be disregarded for reintroduction. Some features were very highly mutated, although they may not be specific to ROS stress. To further understand the specificity of a mutated genetic feature to a condition, the frequency of mutation for a genetic feature across other ALE experiments can be investigated (Figure 2B). From this, frequently mutated features that were also common in other ALE experiments can be disregarded (*rpoB*, *rph*, *pyrE/rph*, *icd*, and *hns/tdk*). Ignoring intergenic regions, this leaves a set highly mutated genes observed in a small set of ALE experiments: *ygfZ*, *aceE, gln X, sucA, pitA,* and *gitA*. Investigating gene functions reveals that almost all of these genes are involved in central and energy metabolism (*aceE, gln X, sucA, and gltA*) or function as transporters (*pitA*). Given that this study aimed to investigate the impact of mutations through gene expression using iModulon analysis, *ygfZ* was selected because of its unknown regulatory potential and its hypothesized role in Fe–S cluster synthesis or repair during oxidative stress.^53^ In summary, *oxyR, fur, iscR,* and *ygfZ* mutants were considered for reintroduction.

To understand if a combination of mutations was feasible, if not more beneficial, the co-occurrence of mutated genes of interest within the same strain was investigated. Most samples exposed to paraquat had more than one gene of interest mutated, with some samples having all four (Figure 2B). These results suggest that the genes targeted for mutation are potentially feasible in combination, prompting further investigation of combinatorial mutant effects in this study.

### Meta-analysis of OxyR ALE Mutations and Related iModulon Activities Revealed Mutations for Potential ROS Tolerance

**Figure 3:**
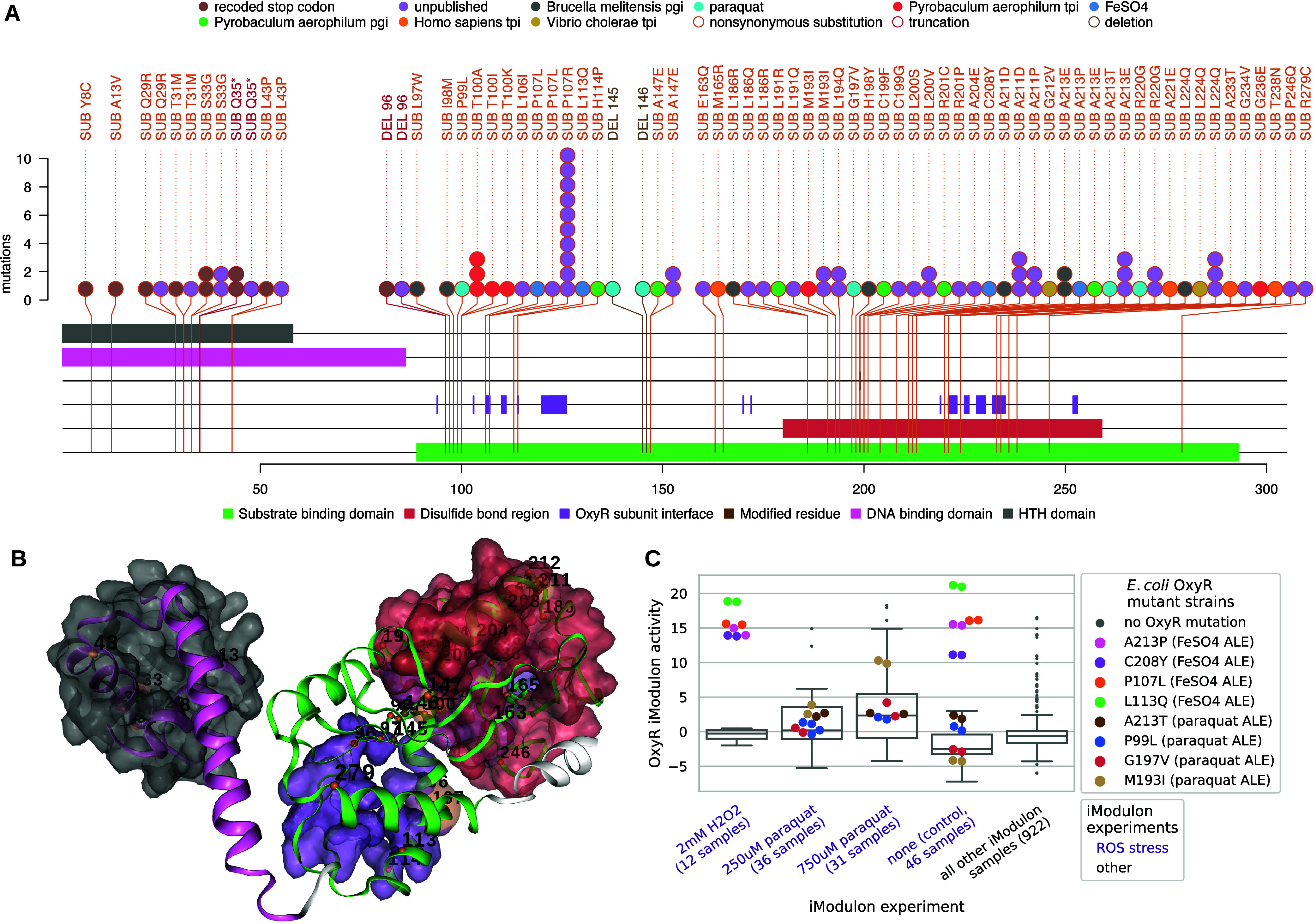
AQALEdb mutations, their effects on OxyR, and to iModulons. (A) Mutation needle plot demonstrating the effect and position of ALEdb mutations to *oxyR*. Slight differences in totals exist between Figure 2B,C and Figures 3 through 6 due to different filtering methods applied (see the ALEdb Mutations section). (B) OxyR’s 3D structure and mutated residues from mutations. The residue chain and transparent surfaces are colored according to the legend of the corresponding mutation needle plot. Mutations are represented by a small opaque sphere with a value representing their amino acid position on the corresponding mutation needle plot. The color of the mutation’s sphere corresponds to the mutation’s predicted effect as described by the legend on the corresponding mutation needle plot. The transparent sphere centered on the mutations’ opaque sphere represents the number of mutations with a specific predicted effect on that position. (C) OxyR iModulon activities for all available samples (1035 from iModulonDB and 12 new samples from this study), where the experiments and strains with *oxyR* mutations are differentiated from the rest of the distribution. The *oxyR* mutant strains were from *E. coli* ALE experiments that manifested *oxyR* mutations as well as mutations to other genes.

*oxyR* mutants were associated with both ALEdb ROS stress conditions of paraquat and FeSO_4_ (Figure 2), and 102 public and unpublished mutations to *oxyR* were extracted from ALEdb for *oxyR* (Figure 3A). Mutations to *oxyR* were proposed to activate ROS protective and preventative functions regulated by OxyR, and strains evolved in conditions involving ROS stressors and hosting *oxyR* mutations had higher growth rates.^4,5^ ALE mutations were found in both of OxyR’s two major domains: the DNA and substrate binding domain (Figure 3A,B). The majority of mutations to OxyR resulted in nonsynonymous substitutions, although mutations resulting in amino acid deletions or premature truncations exist (Figure 3A). The substrate binding domain, hosting the OxyR subunit interface and disulfide bond region that mutations generally cluster around, hosts all of the ROS-related mutations (Figure 3A,B), emphasizing this area as being important for ROS-related selection pressures. An amino acid position in this area, A213, was mutated in both ALEdb ROS-related selection pressures (Figure 3A).

OxyR is a transcription factor regulating genes of the OxyR iModulon, which responds to oxidative stress,^4^ iron homeostasis,^54−57^ and other related environments.^54^ iModulon data were found in iModulonDB for ALE strains hosting the P99L, P107L, L113Q, G197 V, M193I, C208Y, A213P, and A213T OxyR mutations. These strains were from ALE experiments that manifested *oxyR* mutations as well as mutations to other genes. Along with other strains from these ALE experiments, the *oxyR* mutant strains were subjected to the stresses of their original ALE experiment, and their OxyR iModulon activities were determined (Figure 3C). Distributions of *oxyR* mutant and nonmutant strains from these experiments demonstrated increased OxyR iModulon activity relative to the distribution of activities for all other samples. Most *oxyR* mutants fell into the range of OxyR iModulon activity considered outliers for the distribution of all other samples, emphasizing their strong activation of the OxyR iModulon. FeSO_4_ ALE *oxyR* mutants activated the OxyR iModulon across all conditions to which they were subjected; this is possibly due to their unique sequence changes as well as epistasis with other ALE mutations present on the strains. All *oxyR* mutants from the paraquat ALE, except for OxyR A213T, generally increased their OxyR iModulon activity with an increase in paraquat, indicating that A213T was the only mutant with a consistent OxyR iModulon activation.

Overall, the mutation trends and iModulon activity highlight a subset of mutations with possible phenotypic effects. Both ROS ALE experiments selected the OxyR A213 mutations. The A213 mutations were also found in a mutation cluster targeting a functional site on the OxyR sequence that included other ROS ALE mutations. Finally, A213 mutations demonstrated consistent substantial OxyR iModulon activation under all conditions of interest. According to this evidence, both A213T and A213P mutations were proposed for reintroduction (Table S2).

### Meta-analysis of Fur ALE Mutations and Related iModulon Activities Revealed Mutations for Potential ROS Tolerance

**Figure 4:**
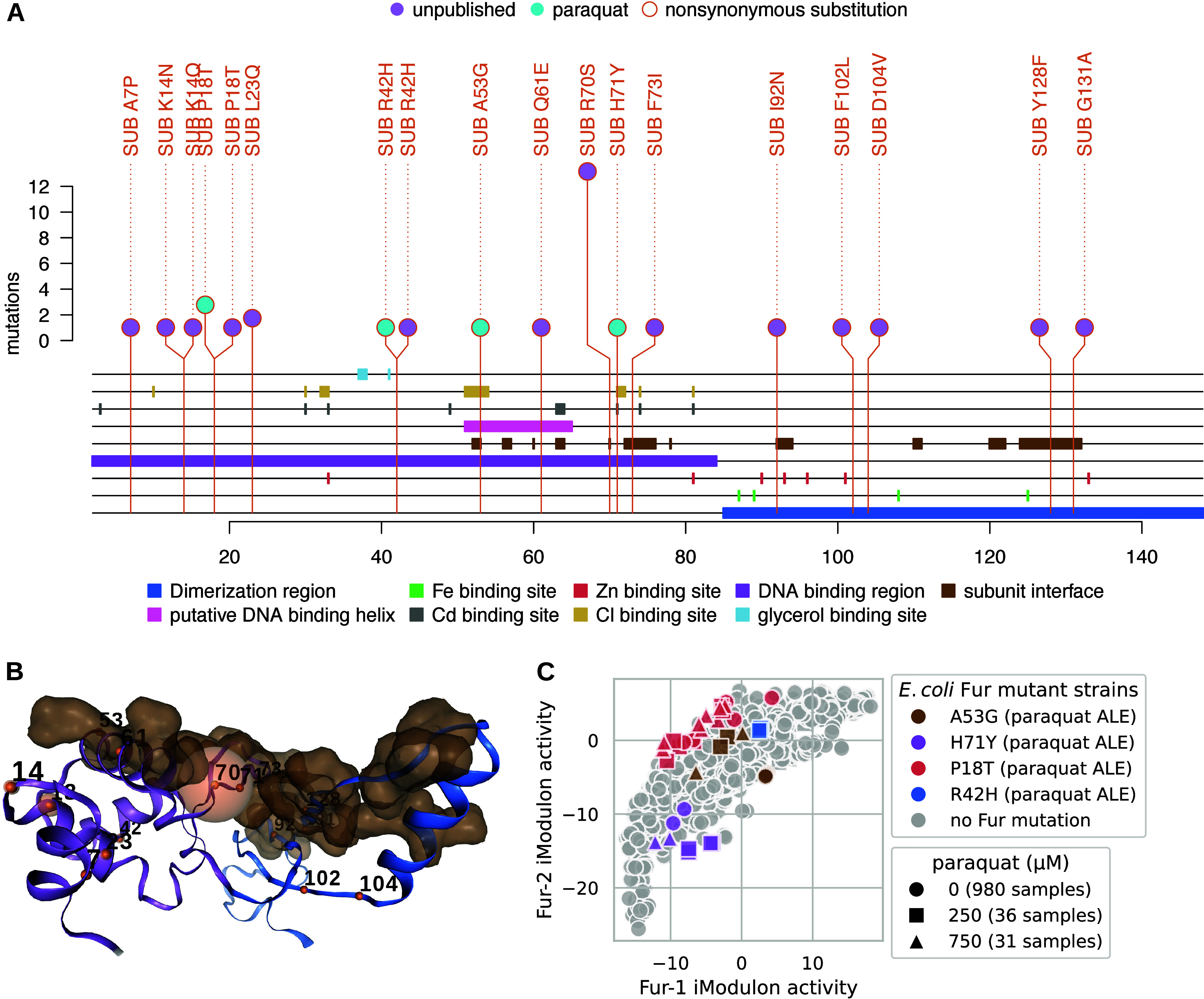
ALEdb mutations and their effects to Fur. (A) Mutation needle plot demonstrating the effect and position of ALEdb mutations to *fur*. Slight differences in totals exist between Figures 2B,C and Figures 3 through 6 due to different filtering methods applied (see the ALEdb Mutations section). (B) Fur’s 3D structure and mutated residues from mutations. The residue chain and transparent surfaces are colored according to the legend of the corresponding mutation needle plot. Mutations are represented by a small opaque sphere with a value representing their amino acid position on the corresponding mutation needle plot. The color of the mutation’s sphere corresponds to the mutation’s predicted effect as described by the legend on the corresponding mutation needle plot. The transparent sphere centered on the mutations’ opaque sphere represents the number of mutations with a specific predicted effect on that position. (C) Fur-1 and Fur-2 iModulon activities for all available samples (1035 from iModulonDB and 12 new samples from this study), where the experiments and strains with *fur* mutations are differentiated from the rest of the distribution. The *fur* mutant strains were from *E. coli* ALE experiments that manifested *fur* mutations as well as mutations to other genes.

*fur* mutants were associated with the ROS stress condition of paraquat (Figure 2), and 39 public and unpublished mutations to *fur* were extracted from ALEdb (Figure 4A). ALE mutations were found in both of Fur’s two major domains (Figure 4A,B). These mutations demonstrated three trends of interest: (1) all of the paraquat mutations are hosted on the DNA binding region, (2) mutations cluster in the first half of Fur’s amino acid sequence, and (3) mutations manifest on or near the subunit interface (Figure 4A). On Fur’s 3D structure, it becomes clearer that mutations to residues 7, 14 18, 23, and 42 comprise a cluster that does not seem to target the subunit interface; besides mutations to residues 102 and 104, the remaining mutations were found on or very close to subunit interfaces (Figure 4B).

Fur regulates two iModulons that are associated with ferric uptake: Fur-1 and Fur-2.^5^ Fur-1 primarily describes systems of siderophore synthesis and transport, whereas Fur-2 describes iron and siderophore transport systems as well as hydrolysis systems.^5^ iModulon data were found in iModulonDB for strains hosting the R42H, P18T, H71Y, and A53G Fur mutations. These strains were from ALE experiments that manifested *fur* mutations as well as mutations to other genes. Along with other end point strains from these ALE experiments, the *fur* mutant strains were subjected to different concentrations of paraquat, and their Fur-1 and Fur-2 iModulon activities were determined (Figure 4C).^5^ Fur-1 and Fur-2 iModulon activities vary across samples, although they demonstrated a trend, where Fur P18T mutations were consistently above the trend, corresponding with a general increase in Fur-2 activity, and H71Y mutations were consistently below the trend, corresponding with a general decrease in Fur-2 activity. P18T is found in the cluster of mutations not targeting subunit interfaces and therefore could represent this set and their potential of decreasing Fur-2 iModulon activity. H71Y is part of the set of mutations landing near or on subunit interfaces and could represent this set and their potential for increasing the Fur-2 iModulon activity. R70S manifests an order of magnitude more than H71Y in ALEdb and may have similar effects. All three of these mutations were proposed for reintroduction to represent all observed trends (Table S2).

### Meta-analysis of IscR ALE Mutations and Related iModulon Activities Revealed Mutations for Potential ROS Tolerance

**Figure 5:**
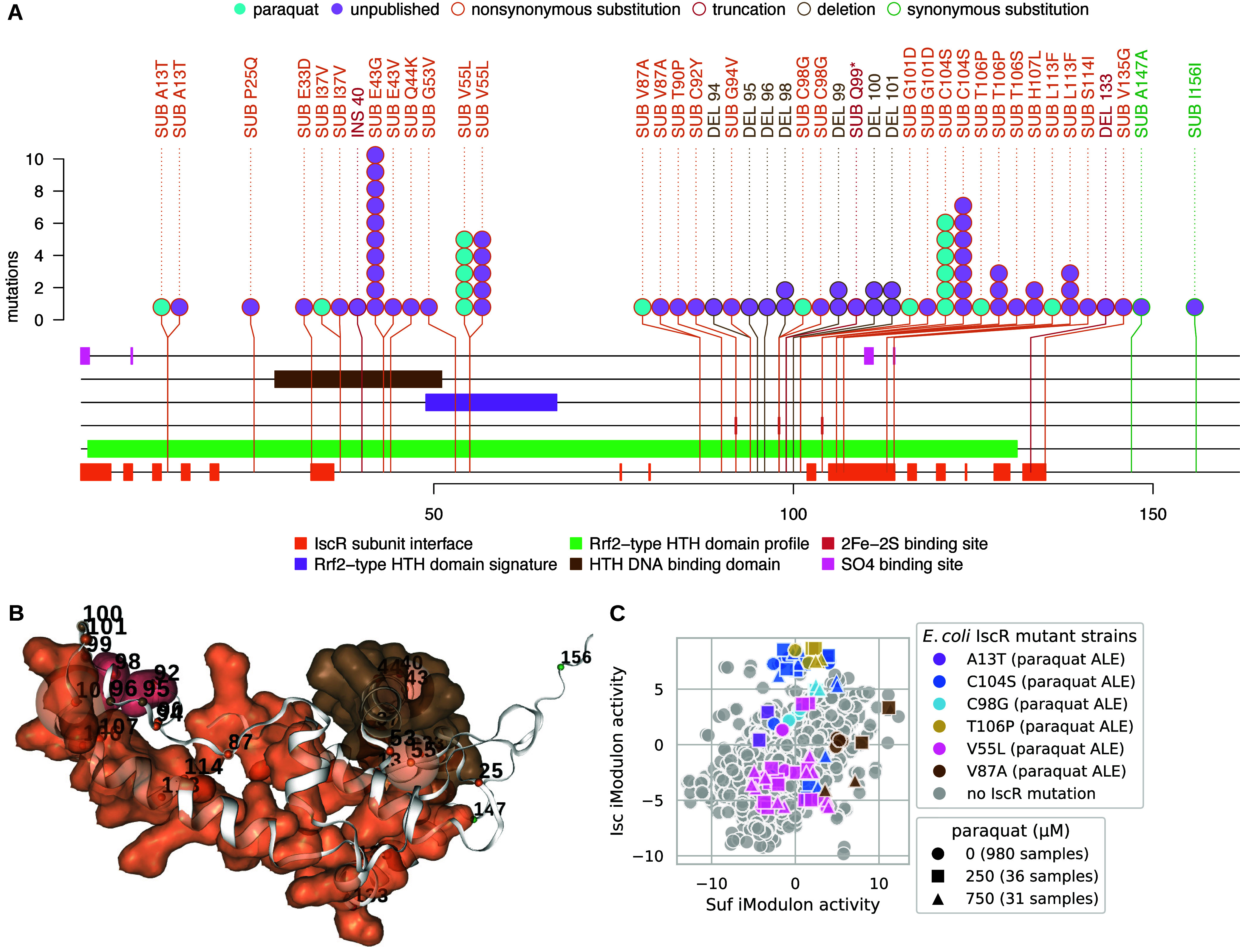
ALEdb mutations and their effects to IscR. (A) Mutation needle plot demonstrating the effect and position of ALEdb mutations to *iscR*. Slight differences in totals exist between Figures 2B,C and Figures 3 through 6 due to different filtering methods applied (see the ALEdb Mutations section). (B) IscR’s 3D structure and mutated residues from mutations. The residue chain and transparent surfaces are colored according to the legend of the corresponding mutation needle plot. Mutations are represented by a small opaque sphere with a value representing their amino acid position on the corresponding mutation needle plot. The color of the mutation’s sphere corresponds to the mutation’s predicted effect as described by the legend on the corresponding mutation needle plot. The transparent sphere centered on the mutations’ opaque sphere represents the number of mutations with a specific predicted effect on that position. (C) Suf and Isc iModulon activities for all available samples (1035 from iModulonDB and 12 new samples from this study), where the experiments and strains with *iscR* mutations are differentiated from the rest of the distribution. The *iscR* mutant strains were from *E. coli* ALE experiments that manifested *iscR* mutations as well as mutations to other genes.

*iscR* mutants were associated with the ROS stress condition of paraquat (Figure 2), and 72 public and unpublished mutations to *iscR* were extracted from ALEdb (Figure 5). ALE mutations most often targeted the 2Fe–2S binding sites, subunit interfaces, and the HTH DNA binding region (Figure 5A,B). Mutation clusters on the 3D structure more clearly demonstrate clustering on or near the 2Fe-2S and the HTH DNA binding domain.

IscR regulates the Isc and Suf iModulons that are both associated with Fe–S cluster synthesis.^5^ iModulon data were found in iModulonDB for strains hosting the A13T, V55L, V87A, C98G, C104S, and T106P IscR mutations. These strains were from ALE experiments that manifested i*scR* mutations as well as mutations to other genes. Along with other end point strains from these ALE experiments, the *iscR* mutant strains were subjected to different concentrations of paraquat, and their Suf and Isc iModulon activities were determined (Figure 5C). Each mutation seemed to correspond with trends in increasing or decreasing activities of either Suf or Isc iModulons. C104S and C98G corresponded to increasing Isc activity. V87A corresponded to increasing Suf activity. A13T corresponded primarily to decreasing Suf activity. Finally, V55L and T106P corresponded to decreased Isc activity and, in the case of V55L, also decreased Suf activity. V55L is also the only mutation with iModulon data found in the cluster near the DNA binding region and HTH domain; all others are found in the cluster near the 2Fe–2S binding sites, SO4 binding sites, or subunit interfaces. For mutations that corresponded to higher Isc iModulon activity, C104S consistently corresponded to the highest activity. V55L consistently corresponded toh the lowest Isc and Suf activity. V87A generally corresponded to the highest Suf activity. These mutations were therefore considered representative for their effects on iModulons and were proposed for reintroduction (Table S2).

### Meta-analysis of YgfZ ALE Mutations and Related iModulon Activities Revealed Mutations for Potential ROS Tolerance

**Figure 6:**
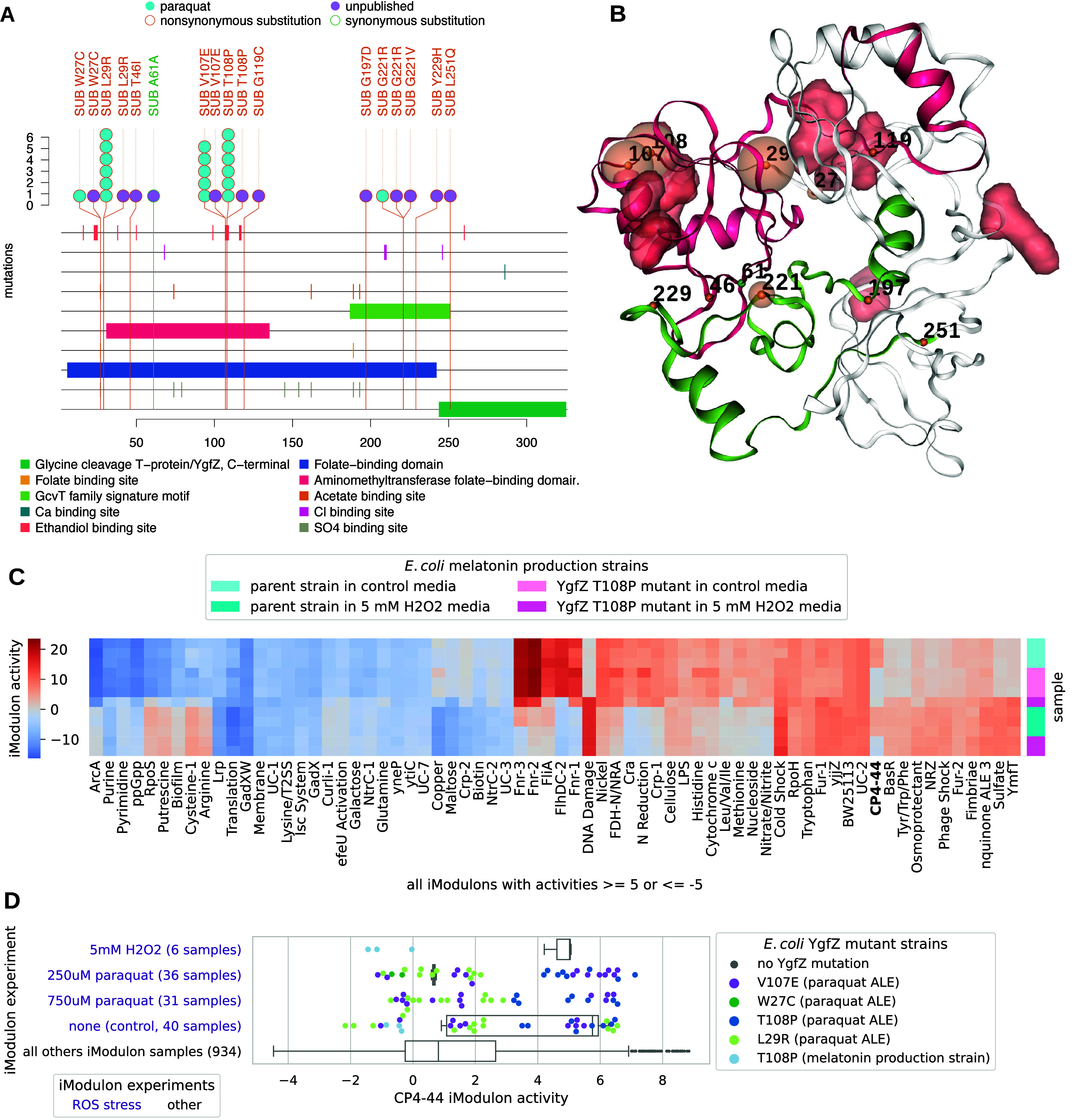
ALEdb mutations and their effects to YgfZ. (A) Mutation needle plot demonstrating the effect and position of ALEdb mutations to *ygfZ*. Slight differences in totals exist between Figures 2B,C and Figures 3 through 6 due to different filtering methods applied (see the ALEdb Mutations section). (B) YgfZ’s 3D structure and mutated residues from mutations. The residue chain and transparent surfaces are colored according to the legend of the corresponding mutation needle plot. Mutations are represented by a small opaque sphere with a value representing their amino acid position on the corresponding mutation needle plot. The color of the mutation’s sphere corresponds to the mutation’s predicted effect as described by the legend on the corresponding mutation needle plot. The transparent sphere centered on the mutations’ opaque sphere represents the number of mutations with a specific predicted effect on that position. (C) Heatmap of iModulon activities for samples from a *ygfZ* mutant characterization experiment using the melatonin production strain as the parent strain. An iModulon activity of ≥5 or ≤−5 is meant to represent a very large change in activity relative to the baseline. (D) CP4-44 iModulon activities for all available samples (1035 from iModulonDB and 12 new samples from this study), where the experiments and strains with *ygfZ* mutations are differentiated from the rest of the distribution. The *ygfZ* mutant strains annotated with “paraquat ALE” were from *E. coli* ALE experiments that manifested *ygfZ* mutations as well as mutations to other genes.

YgfZ is a folate-binding protein that plays a role in Fe–S cluster assembly or repair,^53^ and there is evidence that it is important for oxidative stress resistance (Figure 2A). *ygfZ* mutants were associated with the ROS stress condition of paraquat (Figure 2A), and 46 public and unpublished mutations to *ygfZ* were extracted from ALEdb (Figure 6A,B). ALE mutations generally clustered on or near two functional annotations: (1) the aminomethyltransferase folate-binding domain and (2) the GcvT family signature motif (Figure 6A). On YgfZ’s 3D structure, mutation clusters are more suggestive of two planes, with one plane targeting ethandiol binding sites and the other sitting between all binding sites of the aminomethyltransferase folate-binding domain and the GcvT family signature motif (Figure 6B). Of all these mutations, L29R, V107E, and T108P were most frequently mutated in the paraquat ALE experiment and belonged to two different 1D clusters around the ethandiol binding sites.

To better understand the effects of YgfZ mutations, iModulon activities derived from transcriptional profiles of the melatonin production parent strain with or without the YgfZ T108P were compared under both normal conditions and H_2_O_2_ treatment (Materials and Methods) (Figure 6C). Surprisingly, the YgfZ T108P mutation had little impact on the iModulon activity relative to the presence of H_2_O_2_, although it did coincide with different CP4-44 iModulon activity regardless of environmental conditions. The CP4-44 iModulon, which contains most of the genes for the CP4-44 prophage, was consistently deactivated in the presence of the YgfZ T108P mutation. After this observation, the CP4-44 iModulon activity of these samples was compared to that of all iModulonDB *E. coli* samples with *ygfZ* mutations (Figure 6D). iModulon data were found in iModulonDB for ALE strains hosting the YgfZ L29R, W27C, V107E, and T108P mutations. These strains were from ALE experiments that manifested oxyR mutations as well as mutations to other genes. Along with other strains from these ALE experiments, the *ygfZ* mutant strains were subjected to the stresses of their original ALE experiment, and their CP4-44 iModulon activities were determined. The CP4-44 iModulon activities from iModulonDB and the new samples from this study were combined and compared (Figure 6D). Samples without *ygfZ* mutations demonstrated a broad distribution of CP4-44 iModulon activities (Figure 6D). In *E. coli* ALE end point strains exposed to paraquat, all strains with CP4-44 iModulon of 0 or below had a *ygfZ* mutation except for one without mutations to any gene of interest (Figure S1). Given that *ygfZ* remains an uncharacterized gene, in contrast to the well-studied genes *oxyR*, *fur*, and *iscR* in this study, it is important to determine whether mutations in these other genes also lead to CP4-44 iModulon deactivation: mutations in *oxyR*, *fur*, or *iscR* did not consistently result in CP4-44 iModulon deactivation (Figure S1). The YgfZ L29R and W27C mutations often coincided with reduced CP4-44 iModulon activity (Figure 6D, Figure S1). The YgfZ T108P mutant consistently coincided with reduced CP4-44 iModulon activity in both H2O2 treatment and no treatment when present in the melatonin production parent strain, though not necessarily with ALE-derived strains (Figure 6D, Figure S1). L29R and T108P were therefore chosen for reintroduction because of their high frequency of manifestation in ALE experiments, their location in different mutation clusters, and their evidence of reduced CP4-44 iModulon activities (Table S2).

### ALEdb Mutations Increased Tolerance to ROS-Related Stresses

**Figure 7:**
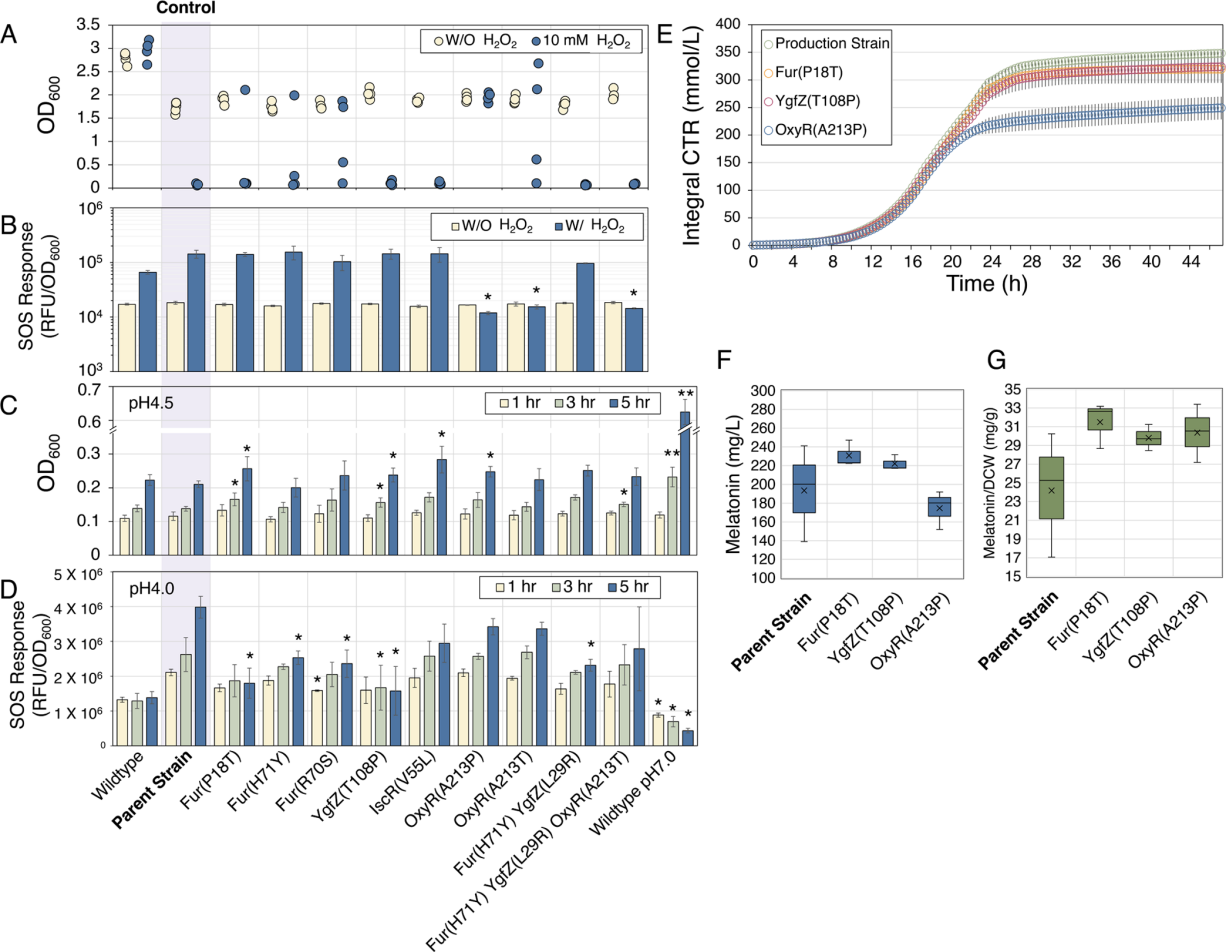
Comparison of growth and stress response between the parent strain (control) and the mutant strains in H_2_O_2_ and acid stress. (A) Biomass represented by OD (600 nm) of the parent strain and strains with ALE mutations implemented with 10 mM H_2_O_2_ treatment (blue) or without (yellow) after 72 h cultivation (Materials and Methods). The dots represent the OD values of each replicate. Whereas the parent strain cannot grow in 10 mM H_2_O_2_, some mutants like OxyR A213P reached the same OD with or without H_2_O_2_. (B) SOS response of wildtype, parent strain, and ALE mutants with or without 10 mM H_2_O_2_ treatment. Data represent the average of three replicates. Error bars indicate standard deviation. Asterisks indicate that the difference is significant (*p* < 0.05) compared to the control (parent strain). (C) Tolerance of the parent strain and ALE mutants in acid stress. Cultures of neutral pH were diluted into pH 4.5. OD (600 nm) was monitored after 1, 3, and 5 h (Materials and Methods). The height of the bars indicates the average concentration of three biological replicates, and error bars indicate the standard deviations. Single asterisk (*) indicates *p* < 0.05, and double asterisk (**) indicates *p* < 0.001, all compared to the control (parent strain). (D) SOS response of the parent strain and ALE mutants in acid stress. Cultures of neutral pH were diluted to pH 4.0. SOS response was monitored after 1, 3, and 5 h using a GFP sensor (Materials and Methods). The height of the bars indicates the average of the three biological replicates. The error bars indicate the standard deviations. Asterisks indicate that the difference is significant (*p* < 0.05) compared to the control (parent strain). (E) Small-scale batch cultivation of the parent strain (control) and strains with one of the three mutations incorporated: Fur P18T, YgfZ T108P, or OxyR A213P. Growth curves represented by integral carbon dioxide transfer rate (CTR) measured online. Data represent the average of three replicates. Error bars indicate standard deviation. (F) Melatonin final titers measured by HPLC after 48 h. (G) Specific melatonin production of four strains normalized by biomass. No statistically significant improvement (*p* < 0.05) in mutant strains compared to the parent.

We constructed nine strains reintroducing single or combinations of ALE mutations (Table S2, Figure 7) using CRISPR-MAD7 or MAGE (Materials and Methods). Combinations were pursued because of the potential for synergistic effects between mutations as evidenced by their co-occurrence in ALEdb clonal samples (Figure 2B). Among all of the mutations, YgfZ L29R and OxyR A213T only appeared in the multiplex MAGE isolates in combination with Fur H71Y. Attempts to construct IscR V87A and IscR C104S using both MAGE and CRISPR-MAD7 were unsuccessful, suggesting that these mutations might cause growth defects in the strain and that their benefits rely on the presence of other ALE mutations. The growth of nine strains was tested in glucose minimal medium with and without H_2_O_2_ (Figure 7A). Whereas the parent strain hardly grew under 10 mM H_2_O_2_, in contrast, a subset of mutants demonstrated more substantial growth. Strains containing Fur P18T, Fur H71Y, Fur R70S, and OxyR A213T mutations had inconsistent growth among the four biological replicates. The strain containing OxyR A213P reached the same OD consistently with or without 10 mM H_2_O_2_, suggesting that this mutation offers superior ROS resistance benefits.

We also examined the SOS response of the strains with ALE mutations. All of the strains were transformed with the SOS reporter plasmid pSD134. The resulting strains were cultivated in glucose minimal media with or without H_2_O_2_ for 24 h. After the treatment of H_2_O_2_, the parent strain showed a very high SOS response compared to the wild-type strain (Figure 7B). Among all the ALE mutations, the strains containing Fur R70S and Fur H71Y + YgfZ L29R demonstrated a reduced SOS response compared to the parent strain. Surprisingly, the three strains containing OxyR A213P and OxyR A213T, either in singleton or in combination with other mutations, did not show an elevated SOS response under the stress of ROS species at all (Figure 7B). This suggested that OxyR A213P and OxyR A213T mutations have activated ROS tolerance machinery and prevented the SOS response caused by H_2_O_2_.

We further tested if the ALE mutations granted any benefits in acid stress, as ROS stress mitigation can benefit acid tolerance. The cells were grown in glucose M9 minimal media at pH 7.0 overnight, and the cultures were diluted into pH 4.5 to OD_600_ 0.1. The OD was monitored after 1, 3, and 5 h. As shown in Figure 7C, the wild-type strain can still maintain the biomass after 5 h exposure to pH 4.5; however, the parent strain has a slight decrease of OD. Mutants containing Fur P18T, YgfZ T108P, IscR V55L, and OxyR A213T had significantly improved survival in low pH.

The effect of ALE mutations on the SOS response during acid stress was also tested (Figure 7D). Similar cultivation experiments were performed using strains containing the SOS sensor plasmid. The strains were grown in a normal M9 glucose medium (pH 7.0) overnight. The cultures were then transferred into the new M9 glucose media at pH 4.0 to OD_600_ 0.05. Samples were taken after 1, 3, and 5 h, and fluorescence was measured. As shown in Figure 7D, all ALE mutations are beneficial in reducing the SOS response during acid stress. Noticeably, strains containing Fur P18T or YgfZ T108P have lower SOS response than other mutants, indicating that these two mutations have a stronger benefit in managing acid stress.

Finally, the melatonin production of the mutants was compared against the parent strain in batch fermentation. Only a subset of strains could be tested (see Materials and Methods); therefore, mutant strains Fur P18T, YgfZ T108P, and OxyR A213P were chosen as representative strains due to each mutant’s consistency in the ROS tolerance experiments (Figure 7A). The results demonstrated that all of the mutants maintained melatonin production (Figure 7F,G). Fur and YgfZ mutants had similar growth curves to the parent strain (Figure 7E) and evidence of better melatonin production (Figure 7F,G), although no statistically significant improvement could be established (*p* < 0.05). The OxyR mutant demonstrated less growth (Figure 7E) and lower melatonin titer than the Fur and YgfZ mutants (Figure 7F), although it did demonstrate higher melatonin production when normalized with biomass (Figure 7G). Also, all mutant melatonin production ranges were more consistent than those of the parent strain.

## Discussion

This study described a meta-analysis workflow leveraging both *E. coli* ALE mutations and iModulon activities to identify a small set of mutations that had evidence of potentially conferring ROS tolerance. Strains incorporating a subset of these mutations were found to have tolerance not only to ROS stress but also to acid stress and reduced SOS responses. These results have several important implications.

First, meta-analysis on aggregated interoperable data types provided valuable evidence for successfully identifying mutations with substantial physiological impact in the presence of ROS stress. The evidence of mutation trends from specific conditions, available through the aggregation of multiple ALE experiment mutations and metadata, enabled the identification of a subset of mutations with potential fitness benefits for these conditions. The inclusion of functional annotations in mutated sequences provided evidence of the gene product functions potentially being targeted by mutation trends, indicating which mutations may result in phenotypic changes. Connecting ALE mutations with iModulon activities proved valuable in interpreting the potential magnitude of a mutation’s impact and provided initial suggestions for their systemic effects. The use of iModulon activities from an aggregated set of experiments provided evidence on whether an iModulon’s activity change is primarily the result of a single mutation or an interplay of multiple elements. The enhanced results due to the interoperable data types enabled screening efforts to focus only on the mutations with the strongest evidence of substantial physiological impact, avoiding the need to screen the much larger set of all available mutations.

Second, most of the selected ALE mutations provided fitness for H_2_O_2_ or acid stress. For H_2_O_2_ stress, Fur and OxyR mutations provided benefit, with OxyR A213P being the only one consistently providing benefit across all replicates. For acid stress, each gene of interest harbored a mutant that consistently contributed significant benefit, with Fur P18T and YgfZ T108P mutations the most beneficial. Although ROS and acid stress responses are expected to be similar,^9−12^ distinct mechanisms likely remain that account for the differences in beneficial mutations. Additionally, single mutants and combinations successfully built in this study may not grant the same benefits as the combinations observed in ALE experiments (Figure 2B) and the absence of mutations to other genes not considered in this study.

Third, mutations engineered into strains for specific optimizations can sometimes have unintended drawbacks on the overall production process. These drawbacks can manifest as a result of the systemic consequences of a mutation, such as resource allocation imbalances, metabolic burden, toxic accumulation of intermediaries, regulatory challenges, pleiotropy, etc. This study’s assays and small-scale batch fermentation results demonstrated that *fur* and *ygfZ* mutations can provide beneficial tolerance, possibly extending operational stability and maintaining production-strain melatonin yield. These results also demonstrated that *oxyR* mutations decreased average melatonin production, emphasizing the possibility of mutations providing a benefit not completely compatible with the primary goal. The OxyR A213P mutation was previously seen to result in the constitutive expression of genes for the ROS scavenging and DNA/protein damage repair system, potentially reducing growth rate due to the introduced metabolic burden.^4^ The Fur P18T mutation was previously expected to upregulate the *feoABC* operon coding for a ROS-sensitive iron transporter.^5^ This was thought to optimize iron uptake for minimizing excess iron-related ROS production^5^ via Fenton reactions.^4^ This strategy may be less of a burden to the cell than the *oxyR* mutations. The YgfZ T108P mutation may enhance its function in Fe–S assembly and repair^53^ relative to the stresses of this study. This enhancement may also be less burdensome than that of *oxyR* mutations. Although these benefits did not translate to increased melatonin titer in the batch fermentation tests, the mutations are believed to offer potential robustness advantages in larger bioreactors where these ROS and acid stresses are more pronounced.^58^ These results also demonstrate how mutations in different genes involved in *E. coli*’s iron utilization in response to oxidative stress can result in different phenotypes

Fourth, this work presented substantial evidence on the potential stress response role of *ygfZ,* a y-gene of currently uncertain function previously linked to Fe–S cluster assembly and repair.^53^ The YgfZ T108P mutation coincided with a decrease in CP4-44 prophage iModulon activity. Prophage gene expression is thought to be triggered by the SOS response,^59^ and the SOS response is expected to be activated in the parent strain as a result of increased melatonin production.^49^ This mutation may enhance YgfZ’s Fe–S assembly/repair function, reducing the SOS response and resulting in CP4-44 iModulon activity. Notably, the YgfZ T108P mutant exhibited the strongest performance among mutants in the fermentation experiment, highlighting its unique and valuable application potential among the mutations studied.

In summary, this study’s findings contribute valuable insights for strain engineering. First, as increasingly large and interoperable data sets become available, meta-analysis approaches leveraging multiple data types will become more valuable for identifying target genes or mutations. Second, meta-analysis identified multiple potential solutions for ROS and acid tolerance, allowing for informed selection of the most advantageous solution, and testing revealed which had drawbacks. Finally, the resulting strains exhibiting a higher stress tolerance and maintaining target production underscore the promise of interoperable data for engineering industrially relevant phenotypes. Overall, this study exemplifies a meta-analysis workflow using interoperable data that is expected to accelerate the engineering of optimized industrial hosts.

## Materials and Methods

### Strain Construction

The *E. coli* strain HMP3427 was derived from a previously reported melatonin production (parent) strain derived from BW25117 (Table S1). All of the ALE mutations were implemented into the genome of HMP3071 (background strain of HMP3427) using one of the following methods. SDT392 and SDT393 were generated by CRISPR/MAD7 as described previously.^30^ SDT711, SDT712, and SDT713 were generated through single target TM-MAGE^60^ where repair oligos contain only one editing target. Other mutants were created using TM-MAGE where repair templates contain a library of oligos. We performed two rounds of transformation of a pool of MAGE oligos (4 μL, premixed in a tube with a total concentration of 100 nmol/mL). Each of the 48 isolates was analyzed by Illumina genome sequencing to validate mutations on the genome. Clones containing single (SDT764, SDT767) or multiple mutations (SDT739, SDT744) were selected for further analysis. All plasmids used in this study were constructed using USER cloning method.^61^

### Growth Test under ROS Stress

The melatonin production plasmid (pHM345) was transformed into each ALE mutant strain by chemical transformation and spread on a Luria–Bertani (LB) agar plate containing 50 μg/L of kanamycin. Following overnight incubation at 37 °C, four colonies of each strain were inoculated in 300 μL of Luria–Bertani (LB) liquid medium supplemented with kanamycin (50 μg/L) in a 96-deepwell plate and incubated at 37 °C overnight with shaking at 250 RPM. Ten microliters of each cultivation was transferred into 250 μL of M9 minimal medium containing 2 g/L of glucose with or without 10 mM H_2_O_2_ and subjected to Growth Profiler (Enzyscreen, Heemstede, Netherlands) to monitor the growth at 30 °C with 250 RPM for 72 h. Growth rates were calculated using the Croissance package.^62^ Kanamycin was not added during cultivation of the control strain DDB35.

### SOS Response Sensor Assay

The SOS response sensor protein was obtained from Addgene (pSMART-SOS-GFPuv, plasmid #102283)^63^ and cloned into a backbone containing p15A origin and chloramphenicol resistance to construct pSD134. Each strain was cotransformed with melatonin production plasmid (pHM345) and pSD134. Strains were cultivated in triplicate into 300 μL of Luria–Bertani (LB) liquid medium supplemented with chloramphenicol (25 μg/L) and kanamycin (50 μg/L) in a 96-deepwell plate at 37 °C overnight. Each 10 μL of broth was transferred into 300 μL of LB medium supplemented with chloramphenicol (25 μg/L) and kanamycin (50 μg/L) in 96-deepwell plates. The plates were incubated in a 37 °C shaking incubator at 250 RPM. At OD_600_ 0.5, H_2_O_2_ (10 mM) was added into one plate, and the fluorescence was monitored after 2 (Figure 1) or 24 h (Figure 7B) (excitation; 485 nm, emission; 520 nm).

### Acid Stress Assay

For SOS response monitoring at low pH, each strain was cotransformed with pHM345 and pSD134. Three independent colonies of each strain were cultivated in 300 μL of M9 minimal medium (pH7.0) containing 2 g/L of glucose, chloramphenicol (25 μg/L) and kanamycin (50 μg/L) for pHM345 containing strains in a 96-deepwell plate at 37 °C overnight. Cells grown overnight were transferred into 400 μL of the same medium adjusted to pH 4.0 in a 96-deepwell plate to start the cultivation at OD_600_ 0.05. The plates were incubated in a 37 °C shaker at 250 RPM. OD_600_ and fluorescence were monitored after 1, 3, and 5 h as described above. For growth monitoring of each strain at low pH, pHM345 was transformed into background strains. Six independent colonies of each strain were picked and inoculated in 300 μL of M9 minimal medium (pH7.0) containing 2 g/L of glucose and kanamycin (50 μg/L) in a 96-deepwell plate at 37 °C overnight. Cells grown overnight were transferred into 400 μL of the same medium adjusted to pH 4.5 to start the cultivation at OD_600_ 0.1 in a 96-deepwell. OD_600_ was monitored after 1, 3, and 5 h cultivated at 37 °C.

### DNA Resequencing

All strains implemented with ALE mutations were sequenced and validated in-house (CfB Biofoundry). For genomic DNA of *E. coli* samples, the CyBio Felix robot and the smart DNA prep (a96)-FX kit (Analytik Jena) were used to extract genomic DNA. The PlexWell 384 kit (SeqWell) was used for tagmentation, barcoding, and library amplification. The final library pool was sequenced with NextSeq. Mutation data were acquired through ALEdb, which uses the breseq mutation finding pipeline.^64,65^ Being that these samples come from different projects, various versions of breseq were used in their mutation data generation. The specific breseq versions for each mutation are documented within ALEdb. The sequencing reads used to generate the mutation data were subjected to quality control through either FastQC (https://www.bioinformatics.babraham.ac.uk/projects/fastqc/) and the FastX-toolkit (http://hannonlab.cshl.edu/fastx_toolkit/) or AfterQC.^66^

### RNA Sample Preparation for iModulon Analysis

RNaseq data were required to investigate substantial changes in all iModulon activities for both the parent strain and the *ygfZ* T108P mutant in the presence and absence H_2_O_2_. HMP3427 and SDT495 were streaked on an LB agar plate containing 50 μg/L kanamycin and incubated at 37 °C overnight. Seed culture was made by inoculating each three colonies into 5 mL (in a 50 mL tube) of M9 minimal medium supplemented with 4 g/L of glucose and 50 μg/L of kanamycin followed by shaking at 250 RPM and 37 °C. OD_600_ of each seed culture was measured after overnight incubation and adjusted to 0.05 into 25 mL of the same medium (in 250 mL baffled flask) to start the main culture followed by incubation at 30 °C and 250 RPM. Each main culture was duplicated to compare the effect of the H_2_O_2_ treatment. Growth was monitored by measuring the OD_600_ until it reached 0.5. Then, 5 mM H_2_O_2_ was added to three flasks of each strain, and the cultures were further incubated for 2 h. Each culture broth was transferred into a new tube containing two volumes of RNAprotect Bacteria Reagent (Qiagen, Hilden, Germany). Samples were pelleted as per manufacturer’s instructions and stored at a −80 °C freezer until extraction. RNAs were extracted using QIAcube (Qiagen, Hilden, Germany) with the RNeasyProtectBacteria/BacterialCellPellts/DNaseDigest protocol. Sequencing of RNA samples was performed by Azenta Life Sciences (GENEWIZ Germany GmbH, Leipzig).

### Generating iModulon Activity Values

The iModulon activity levels in this study were computed and analyzed using an existing iModulon pipeline (github.com/avsastry/modulome-workflow) and analysis toolset (github.com/SBRG/pymodulon) using as input both the complete PRECISE-1K compendium (imodulondb.org/dataset.html?organism=e_coli&dataset=precise1k) and the supplementary samples from this and recent studies.^32,33^ The gene membership of iModulons from the PRECISE-1K compendium was reused.

### Small-Scale Batch Fermentation

The batch fermentation was performed in 10 mL of minimal medium using six-deepwell microplates (Enzyscreen, Heemstede, Netherlands) and incubated at 30 °C with 225 RPM for 48 h using a Kuhner TOM shaker (Kuhner, Birsfelden, Switzerland). The oxygen transfer rate (OTR), carbon dioxide transfer rate (CTR), and respiratory quotient (RQ) were monitored online. HPLC was performed as described previously.^67^ The medium for batch fermentation was described before.^68^ Dry cell weight (DCW, g/L) was calculated by multiplying the final manually measured optical density (600 nm) by a conversion factor of 0.341 (Abs/g_DCW_/L) determined using strain BW25113.

### ALEdb Mutations

The ALE mutations used in this study were from published and unpublished studies. Although all analyses in this study had access to the same full set of ALEdb mutations, each analysis applied different filtering methods on mutations depending on the needs of the analysis. Some filtering methods were applied to mutation sets used in all analyses because they served to generally clean the data to better ensure accurate results. To increase the accuracy of associations by reducing the noise, hypermutator samples were excluded. Hypermutators were defined as samples with at least an order of magnitude more mutations than the other samples within the same ALE experiment. Additionally, all starting strain mutations were removed from the mutation set, and only *E. coli* K-12 MG1655 mutations were used.

Figure 2A,B needed a set of mutations that best represented beneficial mutations for the conditions they manifested in or, in other words, were selected for. Also, being that generating associations exposed the conditions that mutations were manifested in, only public mutations were used so as to reduce the exposure of unpublished data. Mutations with a frequency of 0.5 or higher for a sample were used to ensure that they were likely beneficial, expecting that they had been selected by the selection pressure. For ALEs represented by multiple samples, such as those from different time points, mutations had the potential to appear in multiple samples. To represent unique mutations occurring once per ALE, all mutations across an ALE experiment’s samples were aggregated into a single set, and duplicates were removed.

In Figure 2C, only the common filtering methods were applied. For Figure 2C, this was done to ensure that all samples within an ALE experiment, whether midpoint or end point, were represented.

In Figures 3 and 6, the goal was to maximize the information available to establish trends for likely beneficial mutations. To achieve this, ALE mutations from both published and unpublished studies were included. To protect the anonymity of unpublished mutations, the experimental selection pressures in which they occurred were not disclosed. Common filtering methods were applied. To ensure that the trends represent beneficial mutations, only mutations with a frequency of 0.5 or higher for a sample were used. To represent unique mutations occurring once per ALE, all mutations across an ALE experiment’s samples were aggregated into a single set, and duplicates were removed.

## Data Availability

The software scripts and data supporting the conclusions of this article can be found through the following link: 10.5281/zenodo.11220271

## Supporting Information Available

Strains and plasmids used in this study, the final set of mutations selected for reintroduction, and the CP4-44 iModulon activity levels for iModulonDB strains carrying these mutations of interest (PDF)

## Notes

Grammarly, ChatGPT, Gemini, and Claude.ai were used to refine the narrative and grammar of this work, although these were not used in the research or results interpretation process.

## Abbreviations

ALE: adaptive laboratory evolution
ROS: reactive oxygen species
WT: wild type
AA: amino acid
