# Supplementary material for: Meta-analysis Driven Strain Design for Mitigating Oxidative Stresses Important in Biomanufacturing
Source: ACS Synth Biol. 2024 Jun 27;13(7):2045–59. doi: 10.1021/acssynbio.3c00572 (PMC11264330; doi:10.1021/acssynbio.3c00572)
Supplement: Supplementary file 1 — sb3c00572_si_001.pdf [file sb3c00572_si_001.pdf]

Supporting Information

Title: Meta-Analysis Driven Strain Design for Mitigating Oxidative Stresses Important in Biomanufacturing

Authors: PV Phaneuf[1#*], SH Kim[1#], K Rychel[2], C Rode[1], F Beulig[1], BO Palsson[1,2,3,4], L Yang[1*]

Author Affiliations:
1 Novo Nordisk Foundation Center for Biosustainability, Technical University of Denmark, Kemitorvet, Building 220, 2800 Kongens, Lyngby, Denmark
2 Department of Bioengineering, University of California, San Diego, La Jolla, USA
3 Bioinformatics and Systems Biology Program, University of California, San Diego, La Jolla, USA
4 Department of Pediatrics, University of California, San Diego, La Jolla, CA, USA

# Correspondence

*To whom correspondence should be addressed:

Patrick V Phaneuf
Technical University of Denmark,
Anker Engelunds Vej 1,
2800 Kgs. Lyngby
phaneuf@biosustain.dtu.dk

Lei Yang
Technical University of Denmark,
Anker Engelunds Vej 1,
2800 Kgs. Lyngby
leya@biosustain.dtu.dk

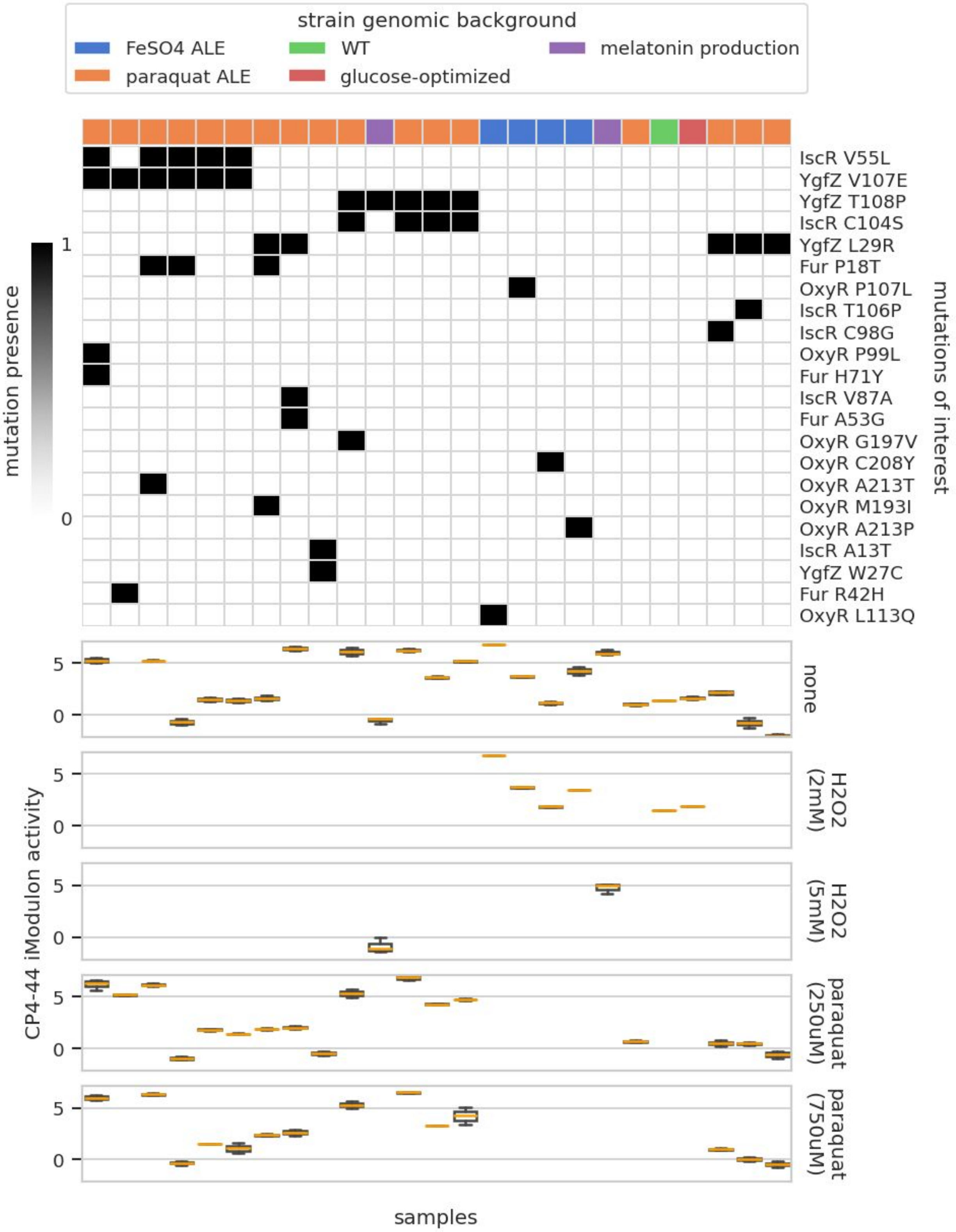

Figure S1. **A comparison between mutation sequence changes to genes of interest across all strains with available iModulon data and their coinciding CP4-44 iModulon activity.**

ID	Genotype	Source
pHM345	P_{J23107}-TpH(E2K, N97I, P99C)-PhhB-ASMT(A258E, V305A), Kan^R	(68)
pSMART-SOS-GFPuv	P_{cda}-GFPuv, Kan^R	(63)
pSD134	P_{cda}-GFPuv, Cm^R, p15A	This work
pGE3	*araC*, P_{ara}-*gam-bet-exo*, tet^R, P_{lac}I-*tetO*, P_{tet}-gRNA_pBR322, P_{j23105}-MAD7, Amp^R, SC101(ts)	(30)
pSD79	P_{j23119}-gRNA_Fur(P18T), Cm^R, pBR322	This work
pSD80	P_{j23119}-gRNA_YgfZ(T108P), Cm^R, pBR322	This work
pMA7	P_{BAD}-*bet-dam*, ColE1, Amp^R	(60)

ID	Genotype	Source
HMP3071	BW25113 Δ*tnaA* Δ*trpR* FolE(T198I) TrpE(S40F) P_{folE}:P_{J23100} Δ*fhuA*::P_2-*ddc* -P_{J23101}-*aanat*	This work. Derived from previous work (49,68)
HMP3427	HMP3071 + pHM345	This work
DDB35	BW25113 Δ*fhuA*	(69)
SDT392	HMP3071 Fur(P18T)	This work

SDT764	HMP3071 Fur(H71Y)	This work
SDT767	HMP3071 Fur(R70S)	This work
SDT393	HMP3071 YgfZ(T108P)	This work
SDT495	SDT393 + pHM345	This work
SDT711	HMP3071 IscR(V55L)	This work
SDT712	HMP3071 OxyR(A213P)	This work
SDT713	HMP3071 OxyR(A213T)	This work
SDT739	HMP3071 Fur(H71Y) YgfZ(L29R)	This work
SDT744	HMP3071 Fur(H71Y) YgfZ(L29R) OxyR(A213T)	This work

Table S1 **Strains and plasmids used in this study**

Gene	Mutation	Reasons for Inclusion	Source
fur	P18T	Hypothesized to increase iron uptake to counteract higher ROS levels	(5)
fur	H71Y	Hypothesized to decrease iron uptake to prevent iron toxicity	(5)
fur	R70S	Unpublished ALEdb mutation near H71Y	Unpublished ALEdb mutation
iscR	C104S	Hypothesized to rebalance Fe-S cluster synthesis and SoxS for better ROS readiness	(5)
iscR	V87A	Hypothesized to rebalance Fe-S cluster synthesis	(5)

		and SoxS for better ROS readiness	
iscR	V55L	Hypothesized to rebalance Fe-S cluster synthesis and SoxS for better ROS readiness	(5)
ygfZ	T108P	Putative Fe-S cluster repair gene; hypothesized to reduce SOS response	(5)
ygfZ	L29R	Putative Fe-S cluster repair gene; hypothesized to reduce SOS response	(5)
oxyR	A213T	A213 mutated in multiple ROS studies	(4,5)
oxyR	A213P	A213 mutated in multiple ROS studies	(4,5)

Table S2. **Table of mutations reintroduced and tested for increased fitness in the presence of ROS agents.**
